# Supplementary material for: Global patterns of vascular plant alpha diversity
Source: Nat Commun. 2022 Sep 1;13:4683. doi: 10.1038/s41467-022-32063-z (PMC9436951; doi:10.1038/s41467-022-32063-z)
Supplement: Supplementary file 1 — Supplementary Information [file 41467_2022_32063_MOESM1_ESM.pdf]

Supplementary Material

To the paper:

Global patterns of local plant species richness

Nature Communications

Francesco Maria Sabatini, Borja Jiménez-Alfaro, Ute Jandt, Milan Chytrý, Richard Field, Michael Kessler, Jonathan Lenoir, Franziska Schrodt, Susan Wiser, Mohammed A.S. Arfin Khan, Fabio Attorre, Luis Cayuela, Michele De Sanctis, Jürgen Dengler, Sylvia Haider, Mohamed Z. Hatim, Adrian Indreica, Florian Jansen, Aníbal Pauchard, Robert K. Peet, Petr Petřík, Valério D. Pillar, Brody Sandel, Marco Schmidt, Zhiyao Tang, Peter van Bodegom, Kiril Vassilev, Cyrille Violle, Esteban Alvarez-Davila, Priya Davidar, Jiri Dolezal, Bruno Hérault, Antonio Galán-de-Mera, Jorge Jiménez, Stephan Kambach, Sebastian Kepfer-Rojas, Holger Kreft, Felipe Lezama, Reynaldo Linares-Palomino, Abel Monteagudo Mendoza, Justin K N'Dja, Oliver L. Phillips, Gonzalo Rivas-Torres, Petr Sklenář, Karina Speziale, Ben J. Strohbach, Rodolfo Vásquez Martínez, Hua-Feng Wang, Karsten Wesche, Helge Bruelheide

Correspondence to: francescomaria.sabatini@unibo.it

Supplementary Methods – Model validation

Our model of local vascular plant richness had relatively high predictive strength. The mean cross-validation, i.e. the correlation between observed and estimated species richness values based on a proportion of withheld data equal to the bag fraction, while still using all data at some stage to fit the model[1], was r = 0.79. When testing the performance of each BRT model comparing the estimated species richness to the observed values for all those plots which were not included in the respective BRT, the average correlation across runs was r = 0.69, with modest variation across iterations (Supplementary Figure 6A). When using spatial block cross-validation, Pearson's r decreased to 0.49. This substantial drop is expected, given the non-independence of points that it corrects, but the underlying signal that it reveals is still strong - far from the situation reported by Ploton et al. [2].

When considering each biome separately, our models had, on average, the highest accuracy in the tropics with year-round and summer rain, and the lowest accuracy in the sub-tropics (Supplementary Figure 6B). Overall, we found no overall bias or trend in the residuals of our

Boosted Regression Tree models, across either grain sizes or biomes (Supplementary Figure 6C, D). Likewise, the spatial distribution of residuals did not show major geographical trends, with the exception of the forest plots in southern Brazil, whose species richness appears to be slightly overestimated (Supplementary Figure 16). We observed no autocorrelation in the model residuals (Supplementary Figure 17).

Supplementary Figure 1

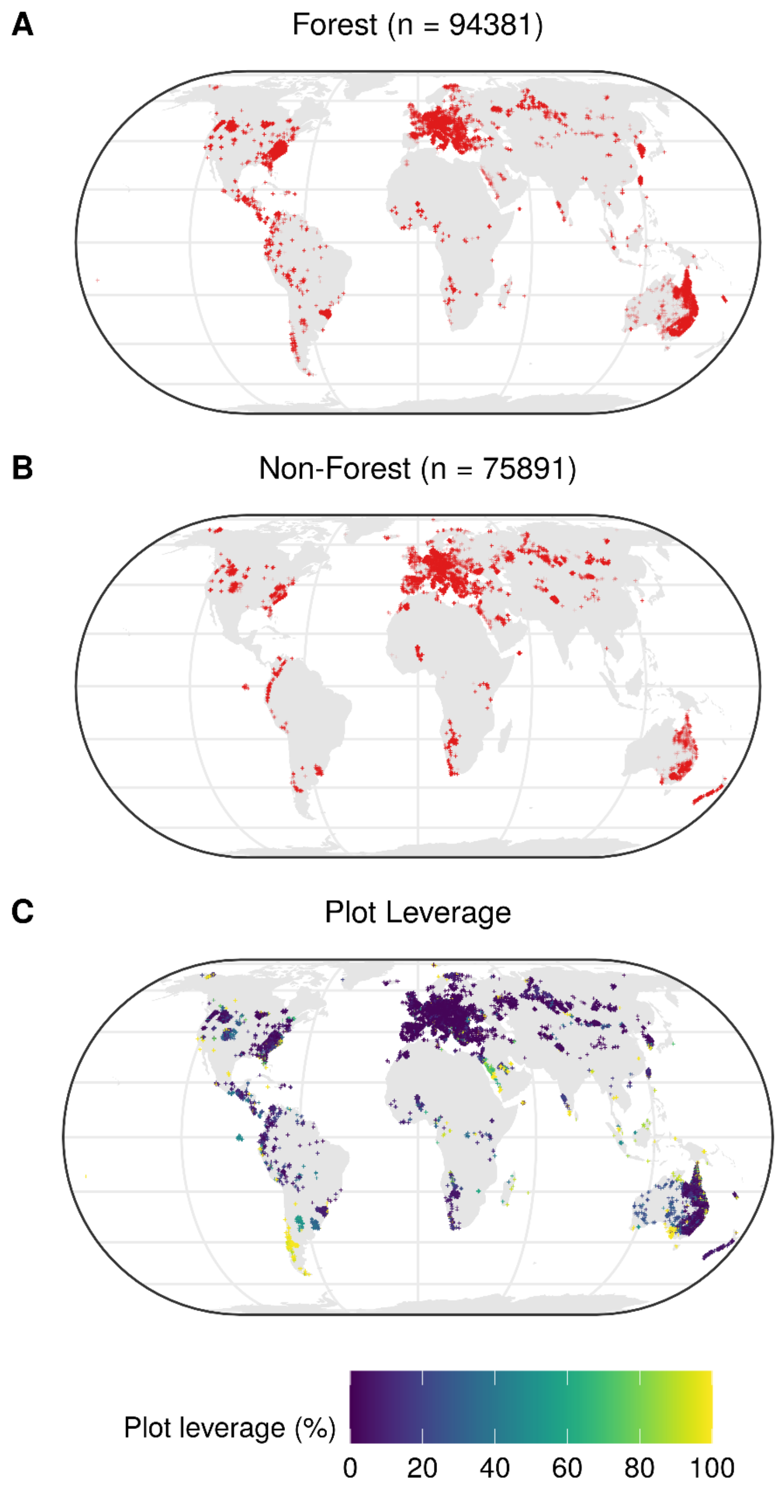

Supplementary Figure 1 – Distribution of vegetation plots used for modelling. Location of all unique forest (A, n = 94,381) and non-forest (B, n = 75,891) plots selected across the 99

stratified resampling iterations; (C) leverage of each vegetation plot, i.e., the percentage of resampling iterations in which a specific plot was selected. Plots with rarer combinations of realm, biome, grain size, and vegetation formation were used in a higher proportion of resampling iterations.

Supplementary Figure 2

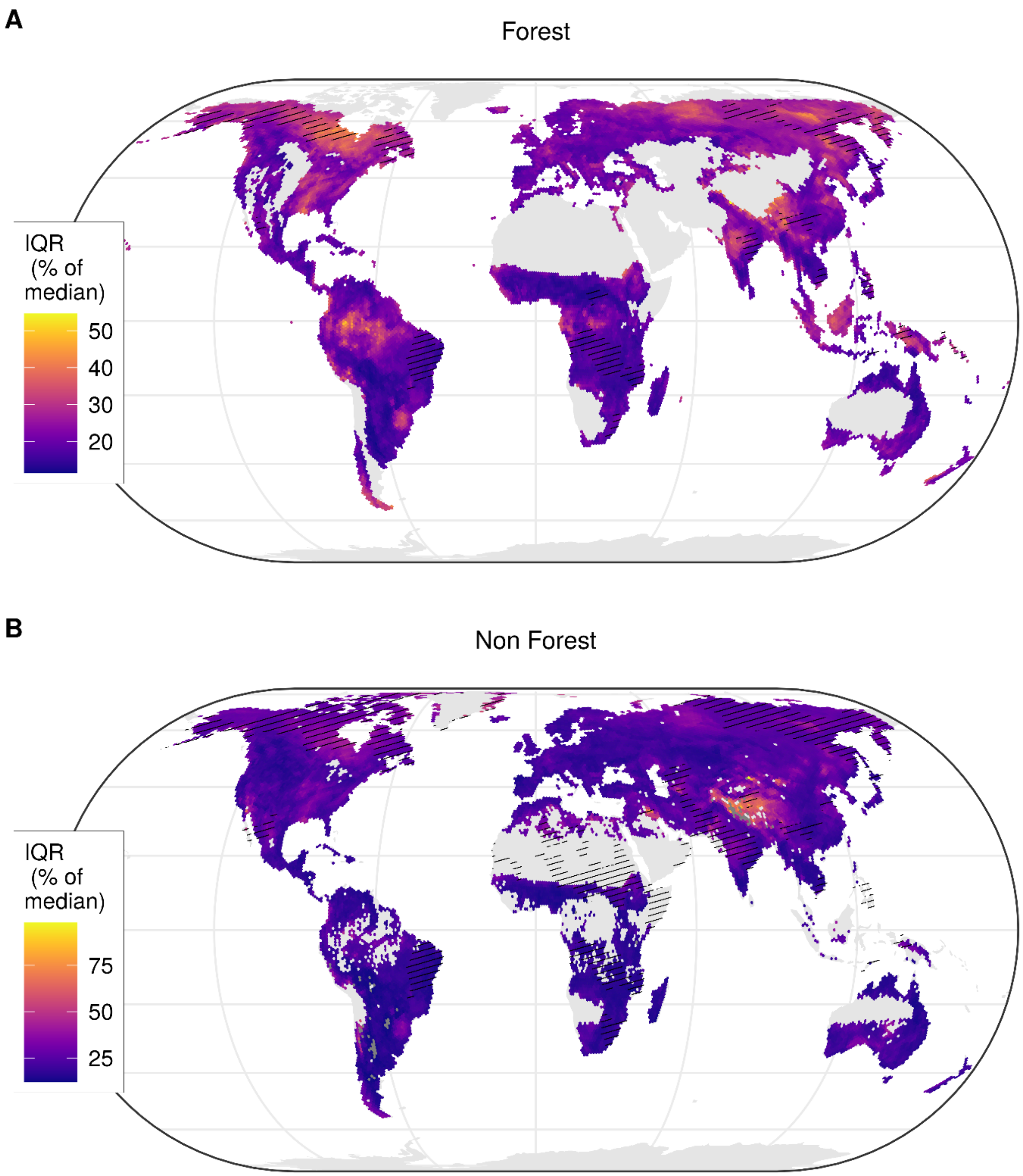

Supplementary Figure 2 – Variability in estimates, measured as the percentage ratio between the interquartile distance (IQR) and the median across 99 resampled subsets. (A) forests (grain = 1,000 m^2); (B) non-forests (grain = 100 m^2). We only show species richness estimates for hexagons where (A) forests would grow under current climate conditions and without human

influence[3], or (B) the land cover ‘herbaceous vegetation’ occurs based on a consensus map, which integrates multiple global remote sensing-derived land-cover products[4]. Parallel hatching represents data-poor regions, i.e., regions farther than 500 km from any sampling plots. Values are averaged over 7,700 km^2 hexagons.

Supplementary Figure 3

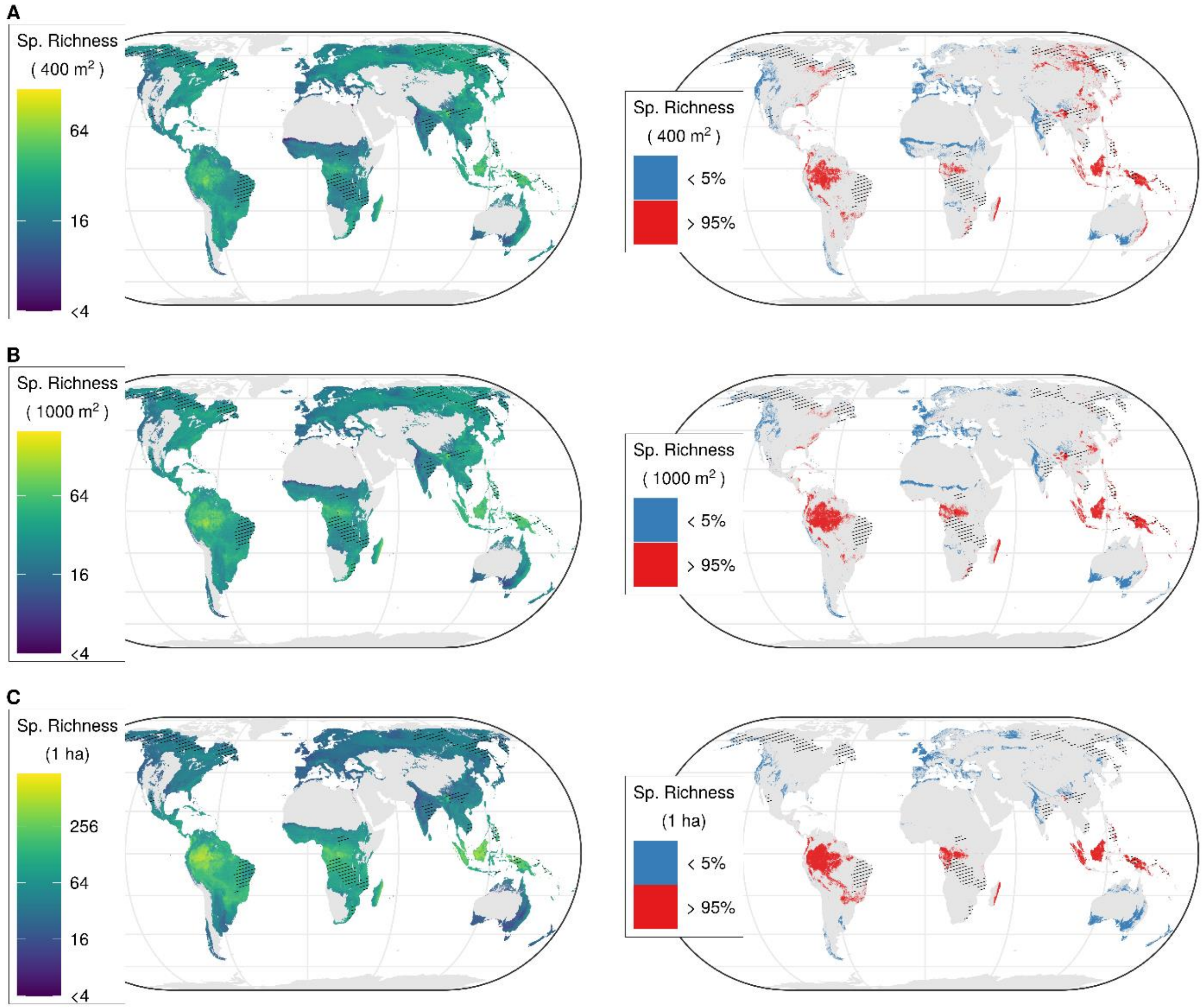

Supplementary Figure 3 – Forests: global distribution of estimated vascular plant alpha diversity at different spatial grains: (A) 400 m2; (B) 1,000 m2; (C) 1 ha. Maps on the left report the median estimated species richness for each 2.5 arcminute grid cell of the World, averaged across 99 boosted regression tree models based on different resampled data sets. Colors are on a log¬2 scale. Maps on the right depict the distribution of hotspots (red) and coldspots (blue), i.e., areas where species richness is above the 95th, or below the 5th global percentile, respectively. We only show alpha diversity estimates for locations where forests would grow under current climate conditions and without human influence31. Hatching represents data-poor regions, i.e., regions farther than 500 km from any sampling plots.

Supplementary Figure 4

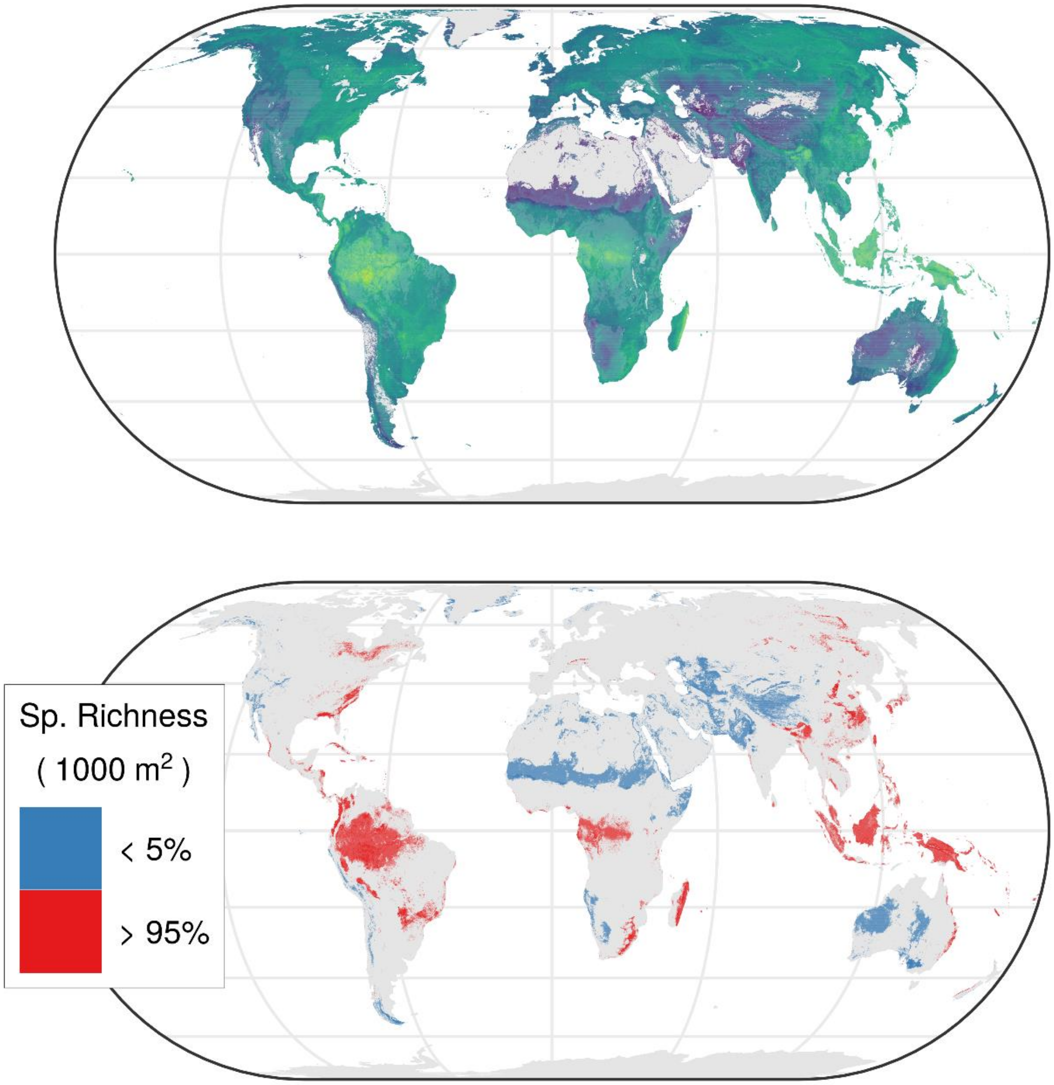

Supplementary Figure 4 – Global distribution of vascular plant alpha diversity across broad formations at 1000 m^2 grain. For each 2.5 arcmin grid cell of the World, the picture shows the species richness of forest ecosystems, if the pixels is within the polygon of potential forest distribution[3], or the species richness of non-forest ecosystems otherwise. In the latter case, we only show species richness estimates for locations where the land cover 'herbaceous

vegetation' based on a consensus map, which integrates multiple global remote sensing-derived land-cover products[4].

Supplementary Figure 5

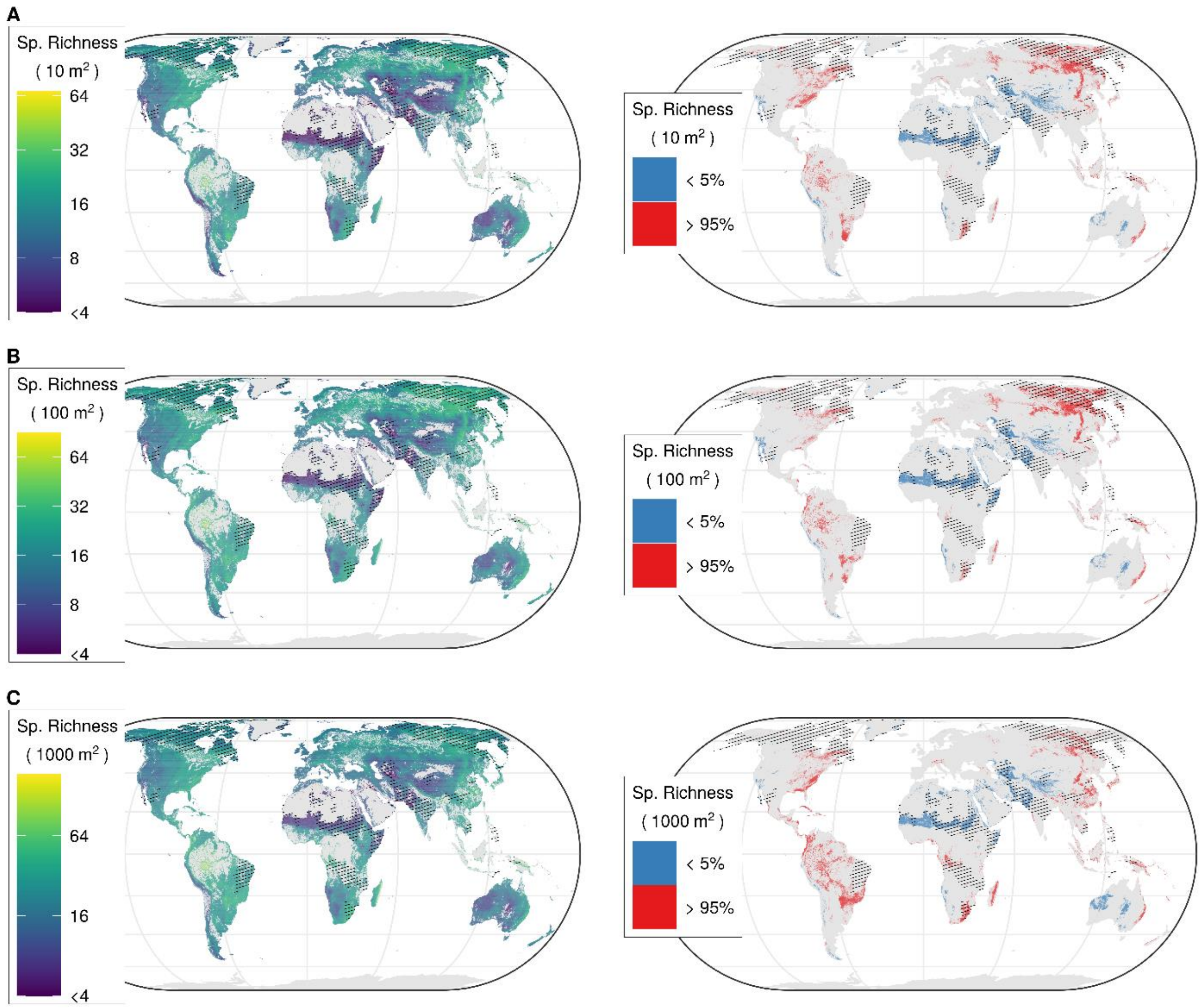

Supplementary Figure 5 - Non-forests: global distribution of estimated vascular plant alpha diversity at different spatial grains: (A) 10 m^2; (B) 100 m^2; (C) 1,000 m^2. Maps on the left report the median estimated species richness for each 2.5 arcminute grid cell of the World, averaged across 99 boosted regression tree models based on different resampled data sets. Colors are on a log_2 scale. Maps on the right depict the distribution of hotspots (red) and coldspots (blue), i.e., areas where species richness is above the 95th, or below the 5th global percentile, respectively. We only show alpha diversity estimates for locations where the land cover 'herbaceous vegetation' occurs based on a consensus map, which integrates multiple global remote sensing-derived land-cover products[4]. Hatching represents data-poor regions, i.e., regions farther than 500 km from any sampling plots.

Supplementary Figure 6

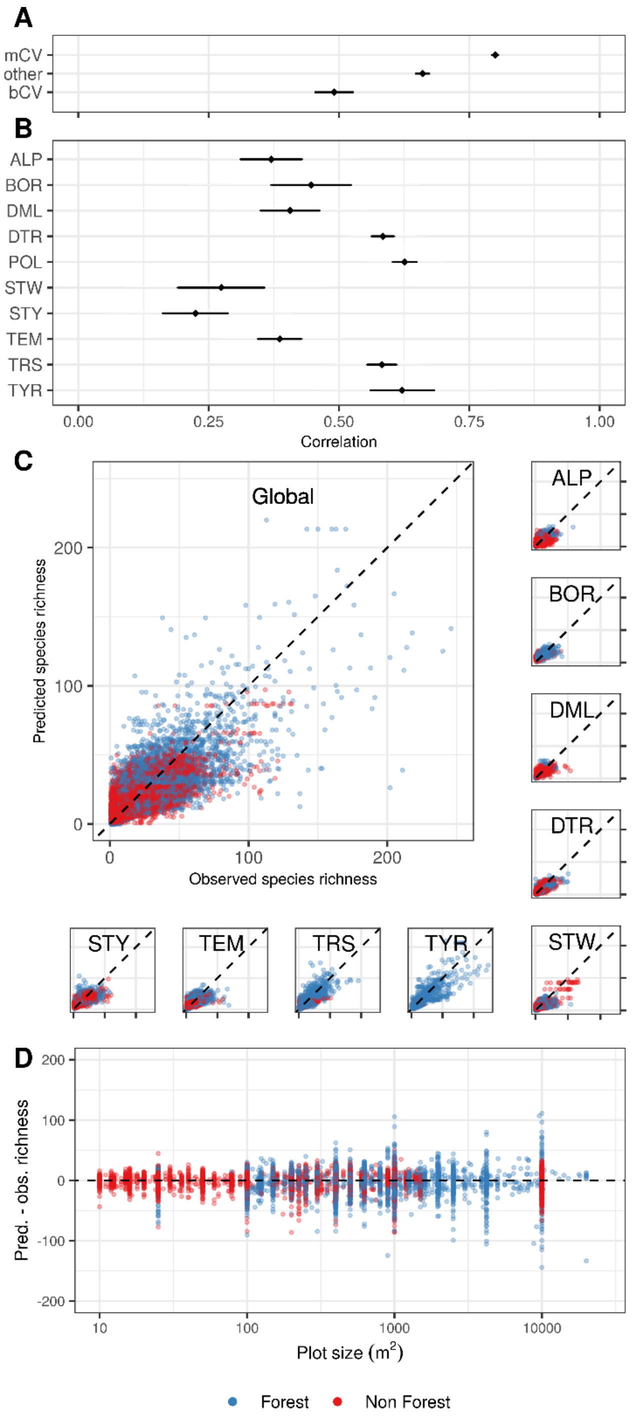

Supplementary Figure 6 – Model and data validation. Cross-validated correlation coefficient of Boosted Regression Trees (BRT) models across 99 iterations for the whole dataset (A), and each individual biome (B). (C) Relationship between predicted versus observed species richness for

the global dataset, and each biome individually. The dashed diagonal line represents a 1:1 relationship between predicted and observed species richness. (D) Distribution of residuals (calculated as predicted – observed species richness) across plot sizes. bCV – block cross-validation, mCV – model cross-validation. ALL – Validation using all plots not included in the model of the respective iteration. Biomes: ALP – Alpine, BOR – Boreal, DML – Dry Mid-Latitudes, DTR – Dry Tropical, STW – Sub-tropical with winter rain, STY – Sub-tropical with year-round rain, TEM – Temperate, TYR – tropical with year-round rain, TRS – Tropical with summer rain, POL – Polar.

Supplementary Figure 7

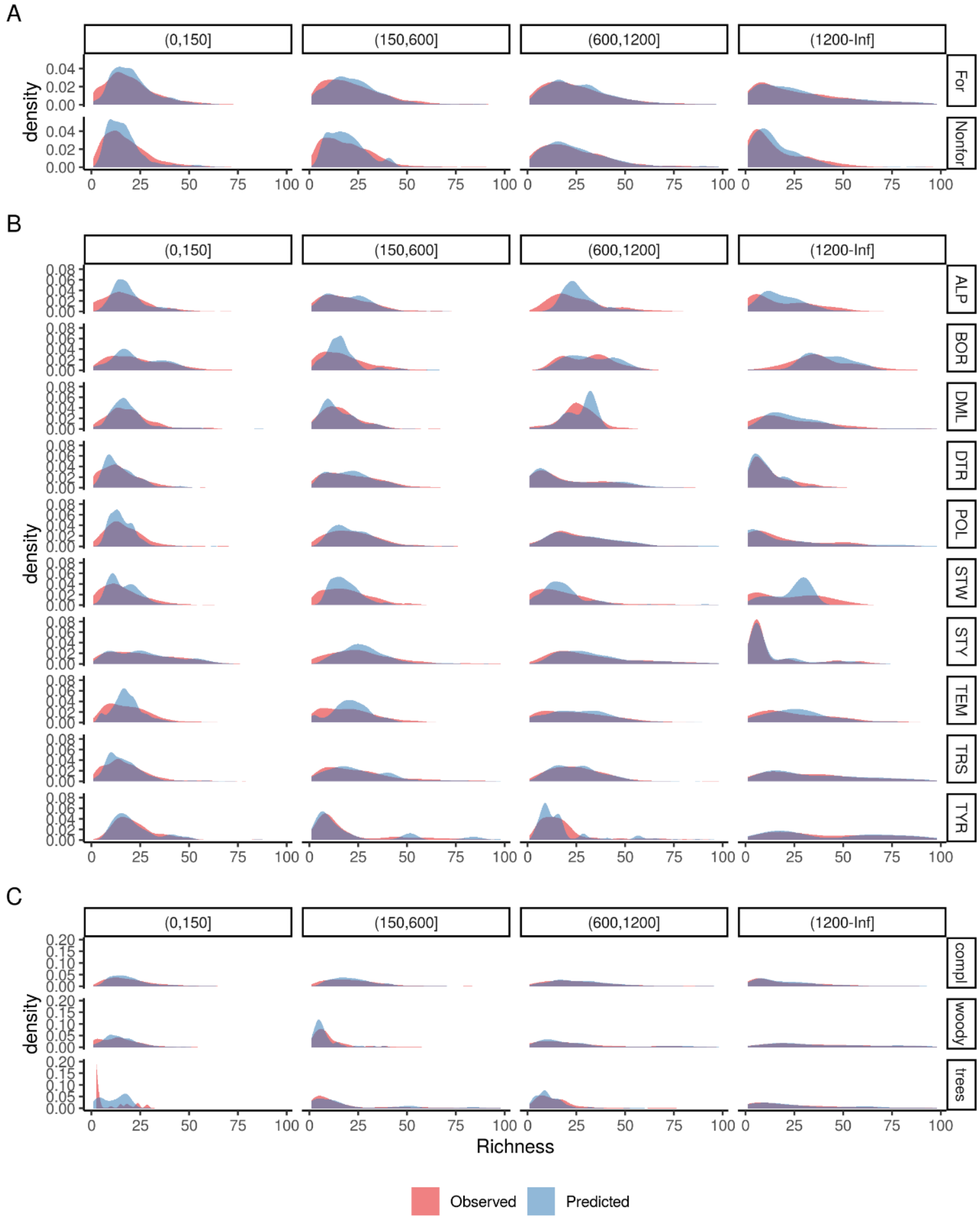

Supplementary Figure 7 – Frequency distributions of observed and predicted local species richness across plot sizes (in m^2) for each broad formation (A), biome (B), and level of data completeness (C).

Supplementary Figure 8

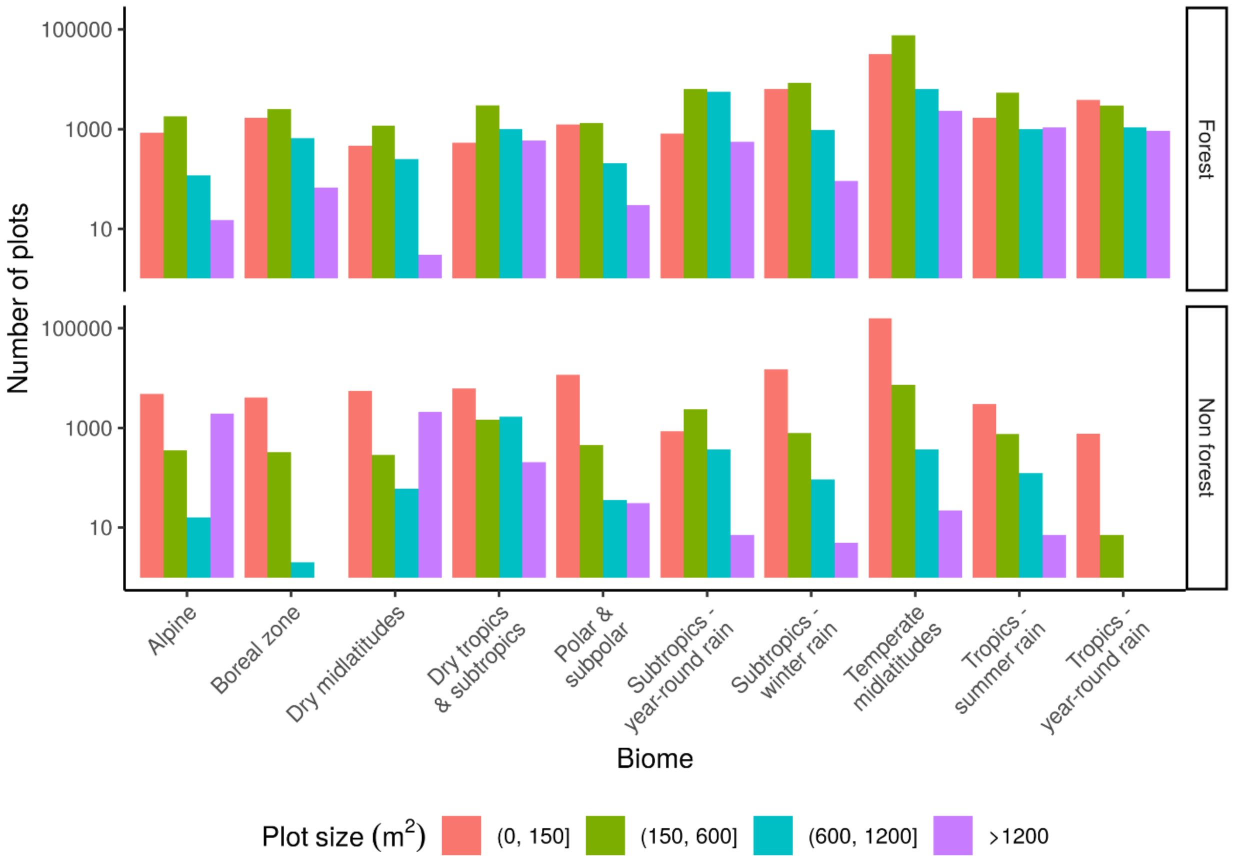

Supplementary Figure 8 – Number of plots in different plot sizes across biomes and formations after data cleaning and bias correction (n = 170,700 plots). The Y-axis is on a log_{10} scale.

Supplementary Figure 9

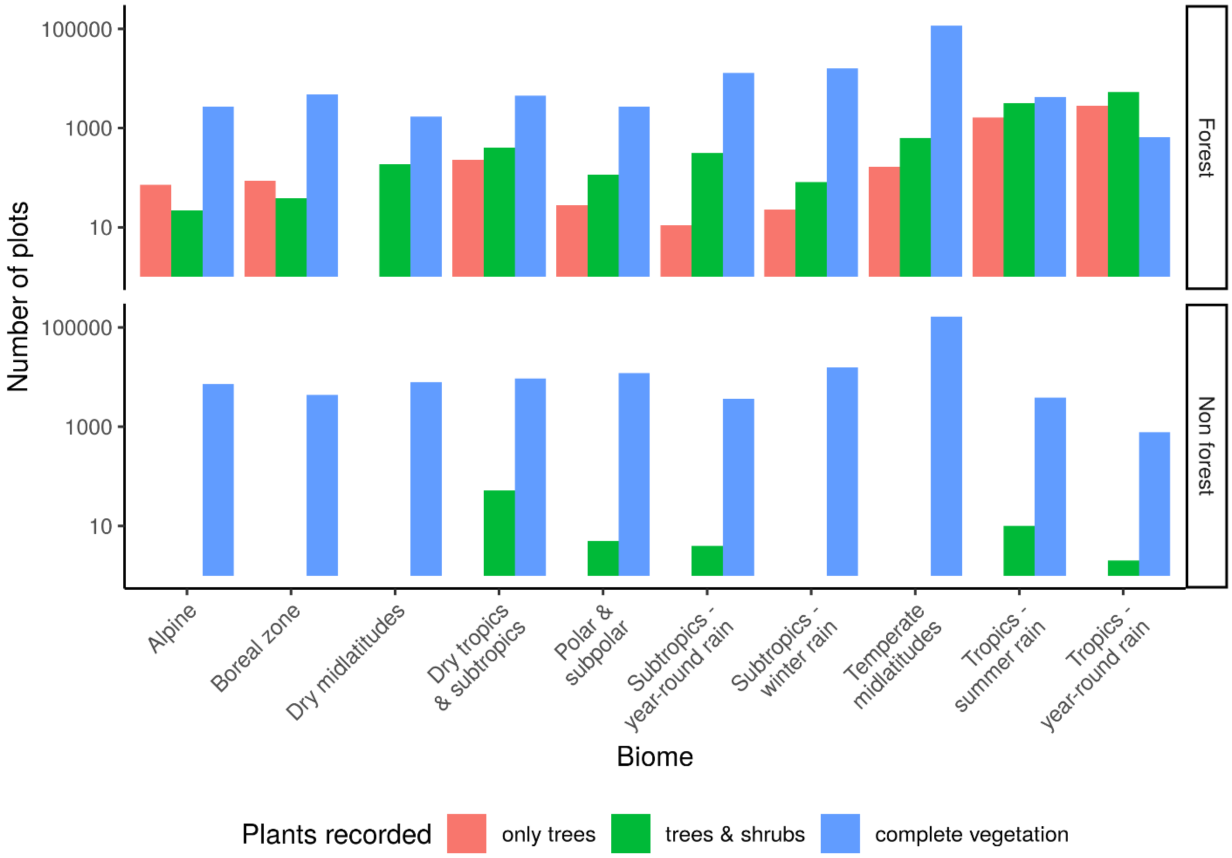

Supplementary Figure 9 – Distribution of plots with different levels of completeness across biomes and broad formation after data cleaning and bias correction (n = 170,272 plots). The Y-axis is on a log_{10} scale.

Supplementary Figure 10

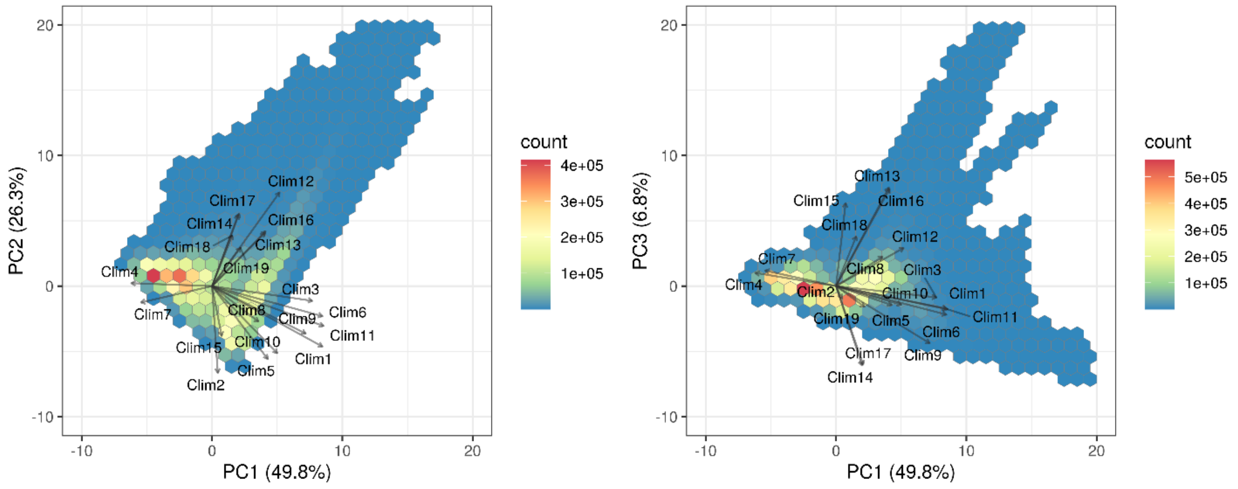

Supplementary Figure 10 – Principal component analysis (PCA) of bioclimatic variables of the world (2.5 arcmin resolution). Values in parentheses represent the variation explained by the respective axis. Color coding represents the density of Earth's pixel with a specific combination of climate conditions. Climate variables derive from [5]. Clim1 = Annual Mean Temperature; Clim2 = Mean Diurnal Range; Clim3 = Isothermality; Clim4 = Temperature Seasonality; Clim5 = Max Temperature of Warmest Month; Clim6 = Min Temperature of Coldest Month; Clim7 = Temperature Annual Range; Clim8 = Mean Temperature of Wettest Quarter; Clim9 = Mean Temperature of Driest Quarter; Clim10 = Mean Temperature of Warmest Quarter; Clim11 = Mean Temperature of Coldest Quarter; Clim12 = Annual Precipitation; Clim13 = Precipitation of Wettest Month; Clim14 = Precipitation of Driest Month; Clim15 = Precipitation Seasonality; Clim16 = Precipitation of Wettest Quarter; Clim17 = Precipitation of Driest Quarter; Clim18 = Precipitation of Warmest Quarter; Clim19 = Precipitation of Coldest Quarter.

Supplementary Figure 11

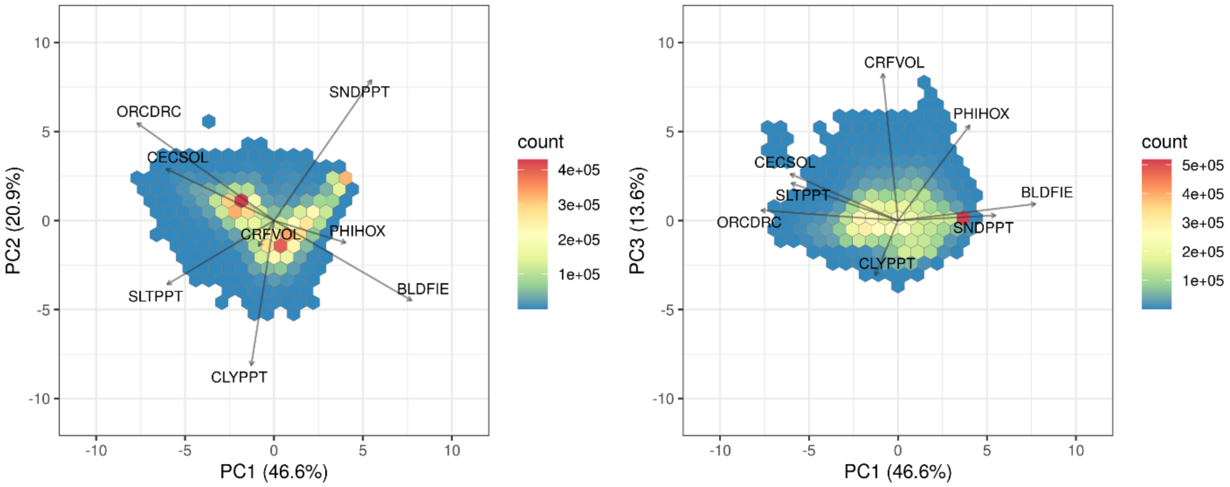

Supplementary Figure 11 – Principal component analysis (PCA) of soil variables of the world (2.5 arcmin resolution). Values in parentheses represent the variation explained by the respective axis. Color codes represent the density of Earth's pixel with a specific combination of soil conditions. Soil variables derive from [6]. CRFVOL = Coarse fragments volumetric in %; SNDPPT = Sand mass fraction in %; SLTPPT = Silt mass fraction in %; CLYPPT = Clay mass fraction in %; BLDFIE = Bulk Density (fine earth) in kg/m^3; CECSOL = Cation Exchange capacity of soil; ORCDRC = Soil Organic Carbon Content in g/kg; PHIHOX = Soil pH x 10 in H_2O.

Supplementary Figure 12

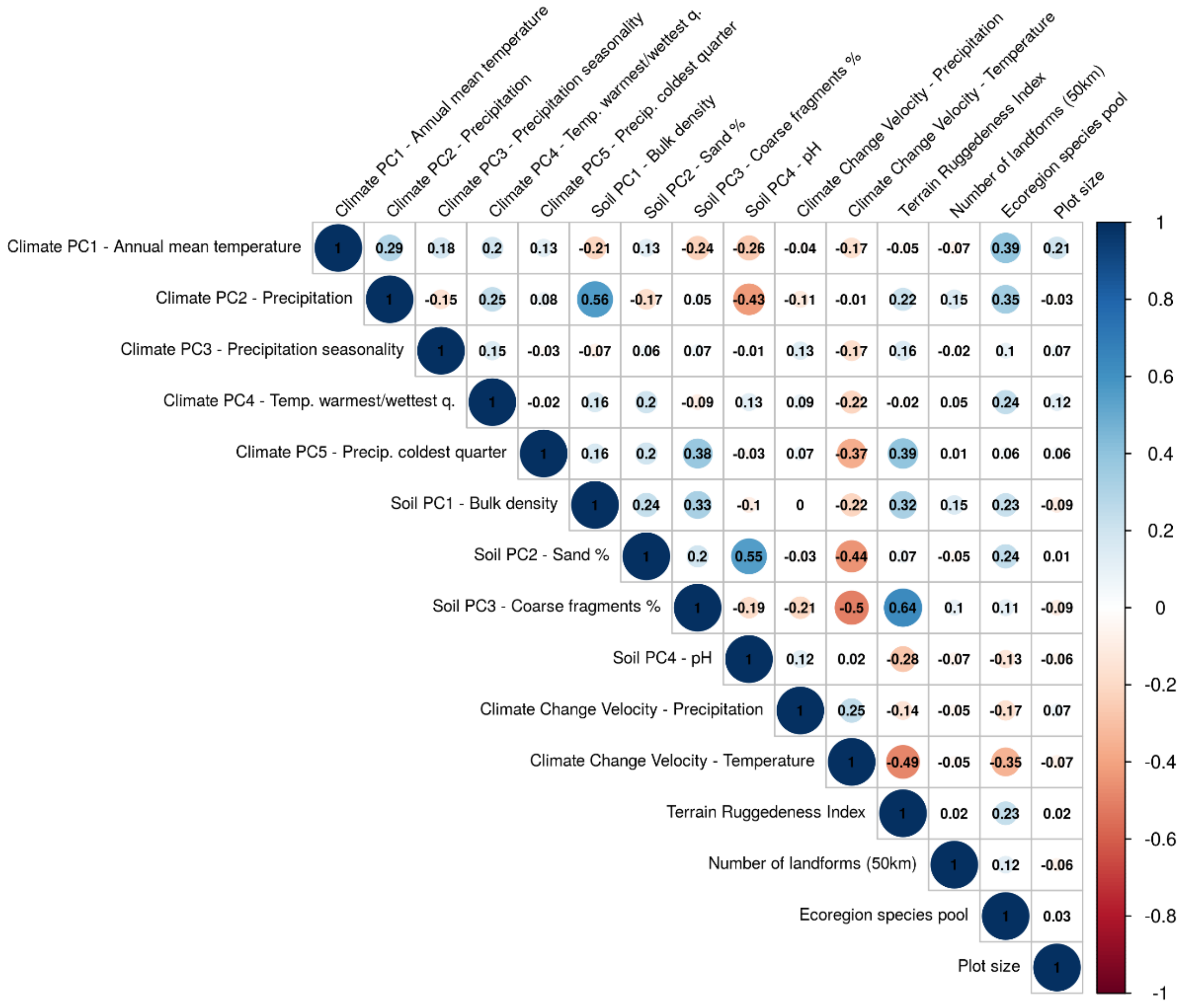

Supplementary Figure 12 – Correlation coefficients of the macroecological and biogeographical gradients used as predictors in boosted regression trees. The color and size of the circles represent the strength of the correlation (Pearson's *r*).

Supplementary Figure 13

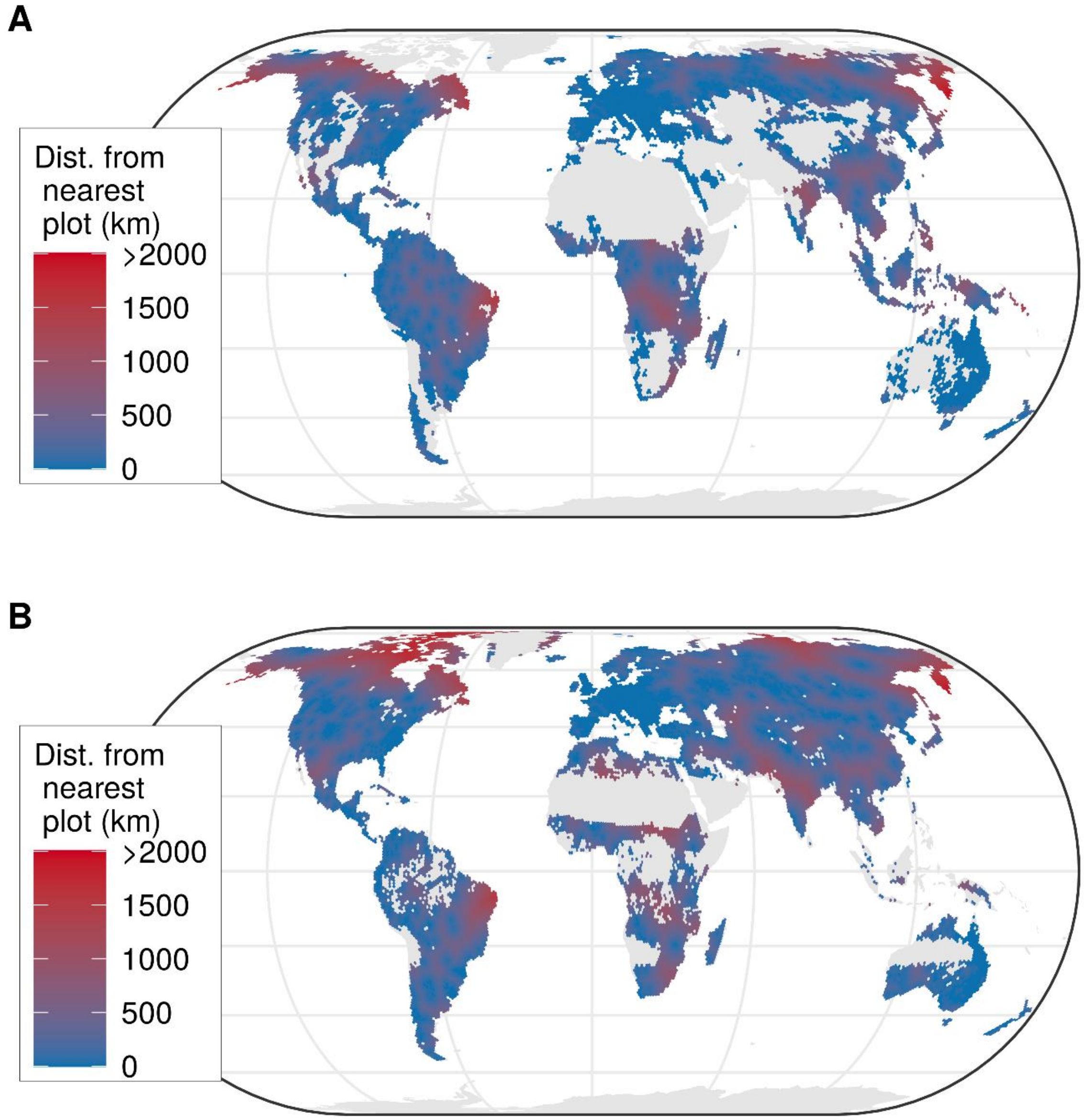

Supplementary Figure 13 – Map of ignorance – distance from the nearest plot used in the analyses in forests (A) and non-forest (B) ecosystems. The size of the hexagons is 7,700 km^2.

Supplementary Figure 14

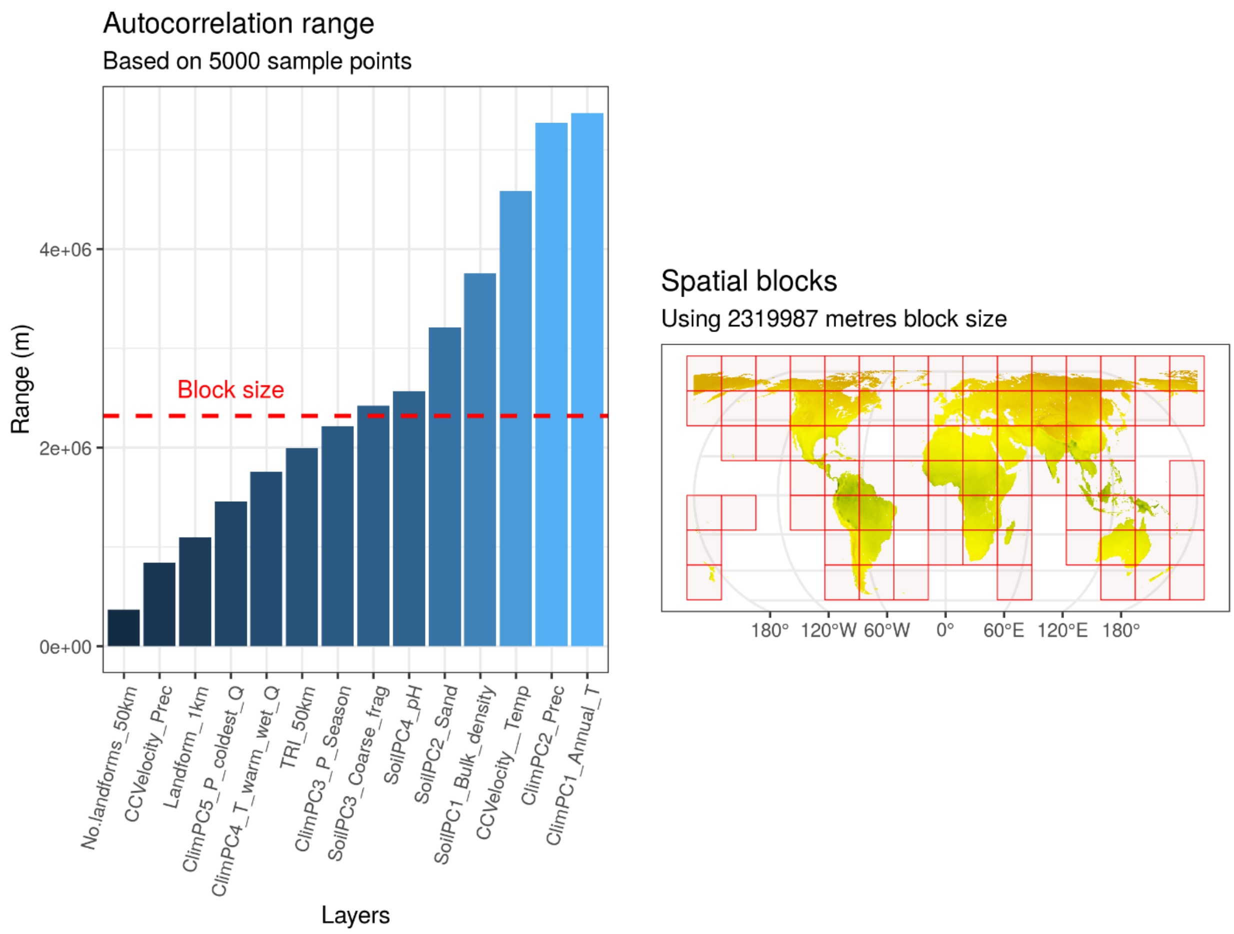

Supplementary Figure 14 – Spatial autocorrelation range of model predictors and corresponding spatial blocks. Block size is based on median spatial autocorrelation range across all spatial predictors.

Supplementary Figure 15

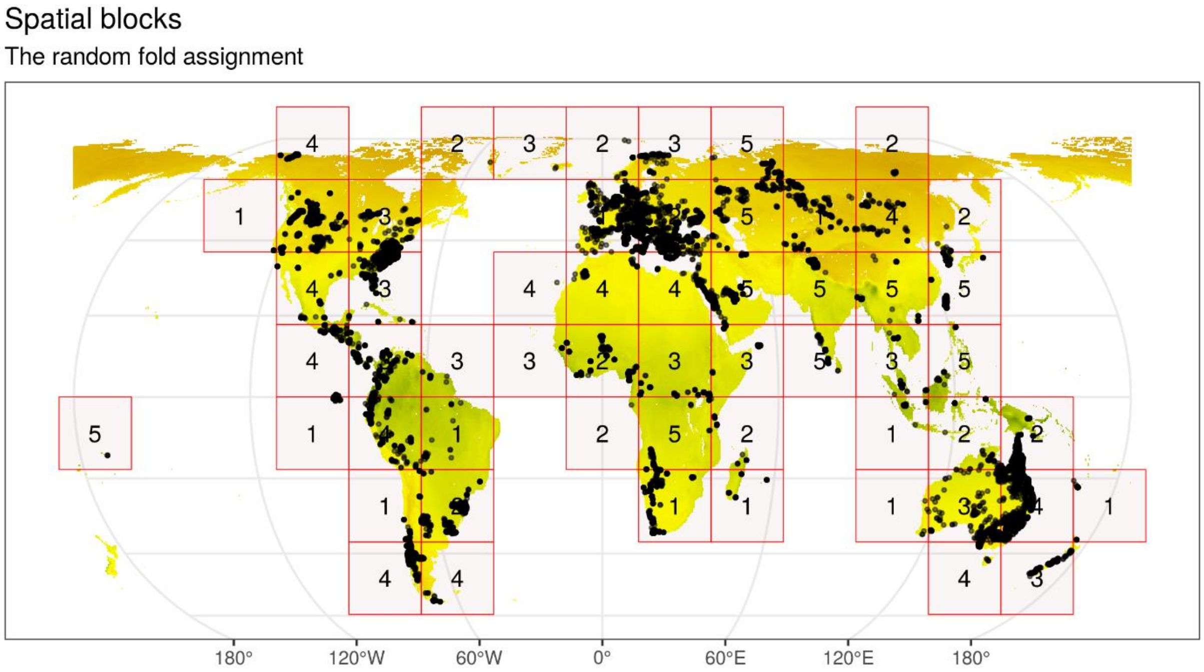

Supplementary Figure 15 – Spatial blocks with random assignment. Each square block has the size of the mean spatial autocorrelation of the spatial predictors. Each block was randomly assigned to one of five folds. Black dots represent data points in the first resampling iteration.

Supplementary Figure 16

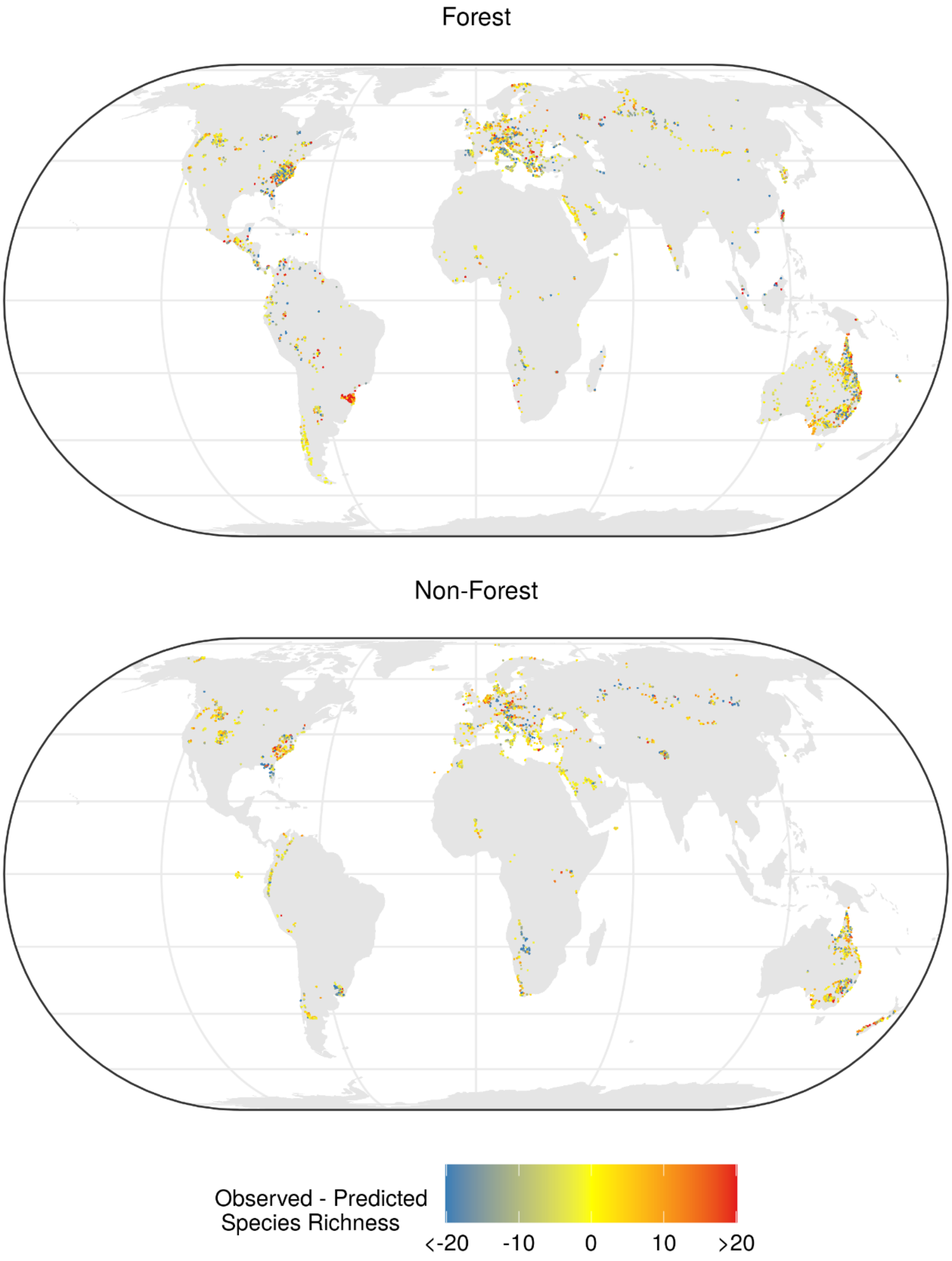

Supplementary Figure 16 – Spatial distribution of the residuals (predicted – observed species richness) of the boosted regression tree based on the first resampling iteration.

Supplementary Figure 17

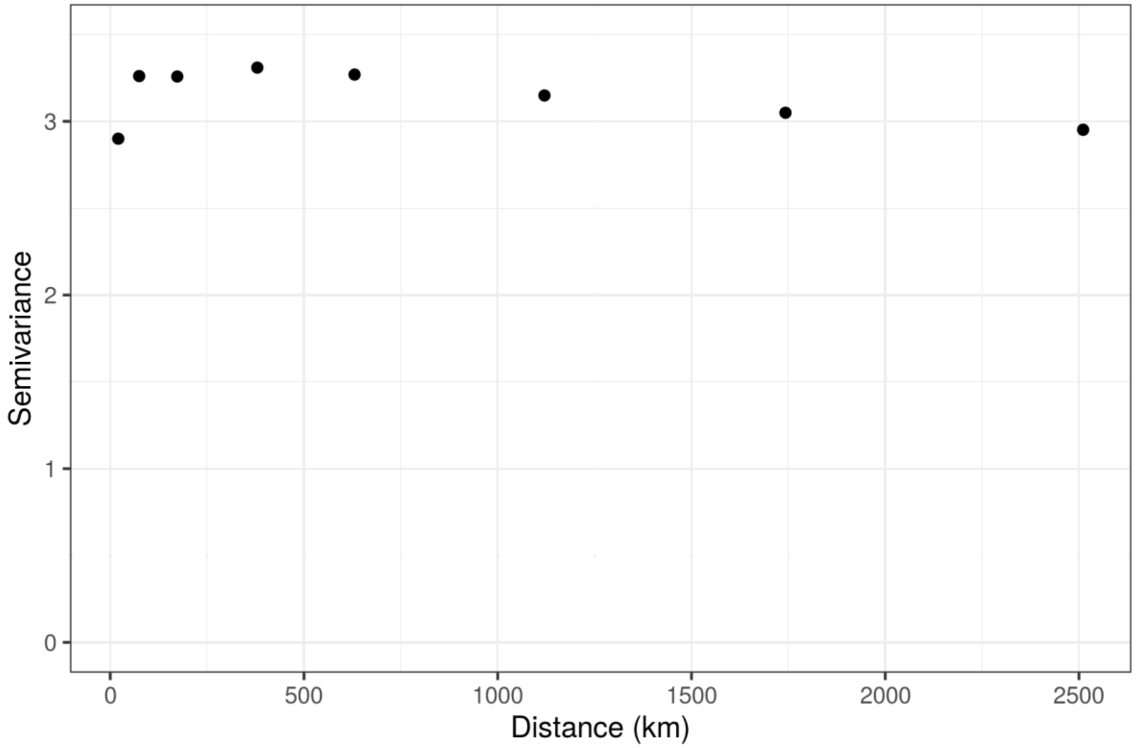

Supplementary Figure 17 – Semivariogram of the residuals of the boosted regression tree based on the first resampling iteration.

Supplementary Table 1

Supplementary Table 1 - Summary of estimated species richness (median across 99 resampled models) at different grains in each biome and globally. Forest and non-forest ecosystems separately. IQR = Interquartile Range.

	**Grain = 400 m²**				**Grain = 1000 m²**				**Grain = 1 ha**			
Forest	**min**	**med**	**max**	**IQR**	**min**	**med**	**max**	**IQR**	**min**	**med**	**max**	**IQR**
Alpine	1	22	79	9	1	24	108	9	2	30	283	10
Boreal zone	4	25	57	8	5	29	75	10	8	38	125	12
Dry midlatitudes	3	23	60	8	5	28	93	10	7	34	99	12
Dry tropics and subtropics	1	20	73	10	2	24	100	10	3	36	328	28
Polar and subpolar zone	5	18	40	7	4	22	47	8	5	29	69	11
Subtropics with winter rain	3	12	47	4	3	16	63	5	5	22	164	7
Subtrop. with year-round rain	6	24	60	8	6	33	108	13	6	38	209	22
Temperate midlatitudes	1	22	77	8	1	25	117	11	3	28	325	12
Tropics with summer rain	1	19	91	7	3	28	126	11	7	80	450	57
Tropics with year-round rain	3	29	120	15	4	47	197	23	5	157	921	128
Global	1	22	120	10	1	29	197	13	2	40	921	39

	**Grain = 10 m²**				**Grain = 100 m²**				**Grain = 1000 m²**			
Non-Forest	**min**	**med**	**max**	**IQR**	**min**	**med**	**max**	**IQR**	**min**	**med**	**max**	**IQR**
Alpine	0	12	39	9	0	17	60	12	0	20	108	13
Boreal zone	2	18	42	7	3	23	55	9	4	28	75	10
Dry midlatitudes	0	11	37	6	0	14	51	7	0	19	93	10
Dry tropics and subtropics	0	9	40	7	0	11	53	8	0	15	100	12
Polar and subpolar zone	2	14	30	5	4	18	39	6	2	21	53	7
Subtropics with winter rain	0	9	29	3	1	12	42	4	1	16	63	5
Subtrop. with year-round rain	2	18	42	6	4	21	50	7	6	31	108	13
Temperate midlatitudes	0	15	39	7	1	18	53	8	2	24	93	12
Tropics with summer rain	0	13	51	4	0	17	67	6	0	27	121	11
Tropics with year-round rain	0	18	68	8	1	24	90	11	2	41	184	19
Global	0	14	68	7	0	17	90	9	0	23	184	13

Supplementary Table 2

Supplementary Table 2 - Summary of observed species richness at different grains in each biome and globally. Forest and non-forest ecosystems separately. IQR = Interquartile Range. Only plots with complete vegetation shown.

	**Grain = (150-600] m²**					**Grain = (600-1,200] m²**					**Grain > 1,200 m²**				
Forest	**n**	**min**	**med**	**max**	**IQR**	**n**	**min**	**med**	**max**	**IQR**	**n**	**min**	**med**	**max**	**IQR**
Alpine	1739	3	19	74	15	120	5	21	73	15	10	14	29	51	26
Boreal zone	2355	1	20	75	13	652	4	34	61	17	66	12	37	82	19
Dry midlatitudes	1001	4	17	88	15	247	7	29	60	9	1	15	15	15	0
Dry tropics and subtropics	2784	1	24	83	20	911	1	14	137	38	375	1	14	45	14
Polar and subpolar zone	1297	1	21	104	14	206	8	29	137	25	16	1	11	51	18
Subtropics with winter rain	6167	2	26	98	17	953	1	21	102	17	76	1	34	58	14
Subtrop. with year-round rain	5722	1	32	135	22	5333	1	36	165	31	500	2	6	89	3
Temperate midlatitudes	15679	1	26	136	21	5935	1	33	171	26	2234	1	31	126	23
Tropics with summer rain	3022	1	34	162	30	547	2	18	137	20	208	1	19	302	41
Tropics with year-round rain	45	5	41	170	20	322	3	27	307	33	130	19	77	839	44
Global	39811	1	26	170	20	15226	1	31	307	27	3616	1	26	839	28
	**Grain = (0-20] m²**					**Grain = (20-150] m²**					**Grain = (600-1,1200] m²**				
Non-Forest	**n**	**min**	**med**	**max**	**IQR**	**n**	**min**	**med**	**max**	**IQR**	**n**	**min**	**med**	**max**	**IQR**
Alpine	1840	1	15	48	11	2725	1	18	72	15	16	10	31	68	10
Boreal zone	77	1	10	42	13	3689	1	28	103	27	2	34	35	35	1
Dry midlatitudes	521	1	10	34	8	4275	1	16	108	14	60	4	21	32	7
Dry tropics and subtropics	1531	1	10	45	8	4381	1	15	100	15	1651	1	36	110	25
Polar and subpolar zone	3515	1	10	49	9	5812	1	12	109	11	36	1	13	49	17
Subtropics with winter rain	2954	1	11	69	11	6155	1	16	108	16	93	1	21	131	73
Subtrop. with year-round rain	22	4	23	31	11	822	1	27	78	36	360	3	22	124	26
Temperate midlatitudes	4594	1	17	95	18	8838	1	20	116	17	368	1	23	119	20
Tropics with summer rain	988	1	12	51	10	1995	1	13	94	12	123	2	31	59	17
Tropics with year-round rain	141	2	13	33	7	628	2	18	50	10	-	-	-	-	-
Global	16183	1	12	95	12	39320	1	17	116	17	2709	1	31	131	27

Supplementary Table 3

Supplementary Table 3 – Correlation coefficients (Pearson's *r*) between climate variables and Principal Component Analysis axes. Underlined values highlight the correlation coefficient with the highest magnitude on a given principal component.

	**PC1**	**PC2**	**PC3**	**PC4**	**PC5**
Clim1 = Annual Mean Temperature	0.84	-0.46	-0.17	0.12	-0.13
Clim2 = Mean Diurnal Range	0.04	-0.58	-0.08	0.39	0.26
Clim3 = Isothermality	0.71	-0.10	-0.08	-0.45	-0.09
Clim4 = Temperature Seasonality	-0.61	0.02	0.10	0.66	0.36
Clim5 = Max Temperature of Warmest Month	0.42	-0.55	-0.14	0.60	0.28
Clim6 = Min Temperature of Coldest Month	0.84	-0.23	-0.22	-0.33	-0.25
Clim7 = Temperature Annual Range	-0.54	-0.12	0.12	0.69	0.41
Clim8 = Mean Temperature of Wettest Quarter	0.31	-0.24	0.20	0.56	-0.30
Clim9 = Mean Temperature of Driest Quarter	0.69	-0.35	-0.42	-0.25	0.07
Clim10 = Mean Temperature of Warmest Quarter	0.49	-0.50	-0.14	0.61	0.16
Clim11 = Mean Temperature of Coldest Quarter	0.85	-0.30	-0.18	-0.28	-0.24
Clim12 = Annual Precipitation	0.51	0.71	0.29	0.19	0.27
Clim13 = Precipitation of Wettest Month	0.40	0.40	0.74	0.03	0.24
Clim14 = Precipitation of Driest Month	0.19	0.53	-0.59	0.46	0.04
Clim15 = Precipitation Seasonality	0.06	-0.32	0.53	0.00	0.32
Clim16 = Precipitation of Wettest Quarter	0.40	0.41	0.73	0.03	0.25
Clim17 = Precipitation of Driest Quarter	0.20	0.54	-0.58	0.46	0.05
Clim18 = Precipitation of Warmest Quarter	0.15	0.37	0.36	0.43	-0.57
Clim19 = Precipitation of Coldest Quarter	0.21	0.28	-0.15	-0.12	0.82

Supplementary Table 4

Supplementary Table 4 – Correlation coefficients (Pearson's *r*) between soil variables and Principal Component Analysis axes. Underlined values highlight the correlation coefficient(s) with the highest magnitude on a given principal component

	**PC1**	**PC2**	**PC3**	**PC4**
BLDFIE = Bulk Density (fine earth) in kg/m^3	0.73	-0.43	0.09	0.29
CECSOL = Cation Exchange capacity of soil	-0.53	0.25	0.23	0.42
CLYPPT = Clay mass fraction in %	-0.12	-0.75	-0.28	-0.27
CRFVOL = Coarse fragments volumetric in %	-0.09	-0.14	0.82	-0.54
ORCDRC = Soil Organic Carbon Content in g/kg	-0.73	0.52	0.05	0.00
PHIHOX = Soil pH x 10 in H_2O	0.39	-0.12	0.52	0.66
SLTPPT = Silt mass fraction in %	-0.55	-0.33	0.19	0.52
SNDPTT = Sand mass fraction in %	0.54	0.78	0.03	-0.25

Supplementary References 1

1 Elith, J., Leathwick, J. R. & Hastie, T. A working guide to boosted regression trees. *J. Anim. Ecol.* **77**, 802-813 (2008).

2 Ploton, P. *et al.* Spatial validation reveals poor predictive performance of large-scale ecological mapping models. *Nat. Comm.* **11**, 4540 (2020).

3 Potapov, P., Laestadius, L. & Minnemeyer, S. *Global map of potential forest cover* www.wri.org/forest-restoration-atlas (2011).

4 Tuanmu, M. N. & Jetz, W. A global 1-km consensus land-cover product for biodiversity and ecosystem modelling. *Glob. Ecol. Biogeogr.* **23**, 1031-1045 (2014).

5 Karger, D. N. *et al.* Climatologies at high resolution for the earth's land surface areas. *Sci. Data* **4**, 170122 (2017).

6 Hengl, T. *et al.* SoilGrids250m: Global gridded soil information based on machine learning. *PLoS One* **12**, e0169748 (2017).
