# Supplementary material for: Global patterns of vascular plant alpha diversity
Source: Nat Commun. 2022 Sep 1;13:4683. doi: 10.1038/s41467-022-32063-z (PMC9436951; doi:10.1038/s41467-022-32063-z)
Supplement: Supplementary file 3 — Description of Additional Supplementary Files [file 41467_2022_32063_MOESM3_ESM.pdf]

Description of Additional Supplementary Files

File Name: Supplementary Data 1
Description: List of plots used from the different data sets composing sPlot. GIVD IDs correspond to those from the Global Index of Vegetation Databases – www.givd.info
