# Supplementary material for: Global patterns of vascular plant alpha diversity
Source: Nat Commun. 2022 Sep 1;13:4683. doi: 10.1038/s41467-022-32063-z (PMC9436951; doi:10.1038/s41467-022-32063-z)
Supplement: Supplementary file 4 — Supplementary Data 1 [file 41467_2022_32063_MOESM4_ESM.docx]

# Supplementary Data 1

To the paper:

Global patterns of local plant species richness

Nature Communications

Francesco Maria Sabatini, Borja Jiménez-Alfaro, Ute Jandt, Milan Chytrý, Richard Field, Michael Kessler, Jonathan Lenoir, Franziska Schrodt, Susan Wiser, Mohammed A.S. Arfin Khan, Fabio Attorre, Luis Cayuela, Michele De Sanctis, Jürgen Dengler, Sylvia Haider, Mohamed Z. Hatim, Adrian Indreica, Florian Jansen, Aníbal Pauchard, Robert K. Peet, Petr Petřík, Valério D. Pillar, Brody Sandel, Marco Schmidt, Zhiyao Tang, Peter van Bodegom, Kiril Vassilev, Cyrille Violle, Esteban Alvarez-Davila, Priya Davidar, Jiri Dolezal, Bruno Hérault, Antonio Galán-de-Mera, Jorge Jiménez, Stephan Kambach, Sebastian Kepfer-Rojas, Holger Kreft, Felipe Lezama, Reynaldo Linares-Palomino, Abel Monteagudo Mendoza, Justin K N'Dja, Oliver L. Phillips, Gonzalo Rivas-Torres, Petr Sklenář, Karina Speziale, Ben J. Strohbach, Rodolfo Vásquez Martínez, Hua-Feng Wang, Karsten Wesche, Helge Bruelheide

Correspondence to: [francescomaria.sabatini@unibo.it](mailto:francescomaria.sabatini@unibo.it)

**Supplementary Data 1** - List of plots used from the different data sets composing sPlot. GIVD IDs correspond to those from the Global Index of Vegetation Databases – [www.givd.info](http://www.givd.info)

| **GIVD ID** | **Dataset name** | **No. of unique plots used** | **Reference** |
| --- | --- | --- | --- |
| 00-00-001 | ForestPlots.net | 1942 | ^1^ |
| 00-00-003 | SALVIAS | 4821 |  |
| 00-RU-001 | Vegetation Database Forest of Southern Ural | 203 |  |
| 00-RU-002 | Database of Masaryk University`s Vegetation Research in Siberia | 1537 | ^2^ |
| 00-RU-003 | Database Meadows and Steppes of Southern Ural | 949 |  |
| 00-TR-001 | Forest Vegetation Database of Turkey - FVDT | 560 |  |
| 00-TR-002 | Non-forest Vegetation Database of Turkey | 2341 |  |
| AF-00-001 | West African Vegetation Database | 1357 | ^3^ |
| AF-00-003 | BIOTA Southern Africa Biodiversity Observatories Vegetation Database | 3164 | ^4^ |
| AF-00-006 | SWEA-Dataveg | 394 |  |
| AF-00-008 | PANAF Vegetation Database | 2126 |  |
| AF-00-009 | Vegetation Database of the Okavango Basin | 590 | ^5^ |
| AF-00-010 | Afroalpine vegetation | 250 |  |
| AF-00-011 | West African Secondary Forests | 105 | ^6^ |
| AF-BF-001 | Sahel Vegetation Database | 808 | ^7^ |
| AF-CD-001 | Forest Database of Central Congo Basin | 292 | ^8^ |
| AF-CM-001 | Cameroon Forest Database | 172 | ^9^ |
| AF-ET-001 | Vegetation Database of Ethiopia | 74 | ^10^ |
| AF-MA-001 | Vegetation Database of Southern Morocco | 1337 | ^11^ |
| AF-NA-001 | National Phytosociological Database of Namibia | 1308 | ^12^ |
| AF-ZA-003 | SynBioSys Fynbos Vegetation Database | 809 |  |
| AF-ZW-001 | Vegetation Database of Zimbabwe | 30 | ^13^ |
| AS-00-001 | Korean Forest Database | 4561 | ^14^ |
| AS-00-003 | Vegetation of Middle Asia | 313 | ^15^ |
| AS-00-004 | Rice Field Vegetation Database | 9 |  |
| AS-BD-001 | Tropical Forest Dataset of Bangladesh | 87 |  |
| AS-CN-001 | China Forest-Steppe Ecotone Database | 73 | ^16^ |
| AS-CN-002 | Tibet-PaDeMoS Grazing Transect | 131 | ^17^ |
| AS-CN-003 | Vegetation Database of the BEF China Project | 27 | ^18^ |
| AS-CN-004 | Vegetation Database of the Northern Mountains in China | 484 |  |
| AS-CN-008 | 100 plots from Ma'anling valcano park in Haikou, China | 472 | ^19^ |
| AS-EG-001 | Vegetation Database of Sinai in Egypt | 273 | ^20^ |
| AS-ID-001 | Sulawesi Vegetation Database | 24 |  |
| AS-ID-002 | EFForTS-CRC990 Sumatra vegetation plots | 160 | ^21^ |
| AS-IN-XXX | Vegetation of Ladakh (India) | 4623 | ^22^ |
| AS-IR-001 | Vegetation Database of Iran | 1112 |  |
| AS-KG-001 | Vegetation Database of South-Western Kyrgyzstan | 442 | ^23^ |
| AS-KZ-001 | Database of Meadow Vegetation in the NW Tien Shan Mountains | 94 | ^24^ |
| AS-MN-001 | Southern Gobi Protected Areas Database | 862 | ^25^ |
| AS-RU-001 | Wetland Vegetation Database of Baikal Siberia (WETBS) | 13 | ^26^ |
| AS-RU-002 | Database of Siberian Vegetation (DSV) | 5547 | ^27^ |
| AS-RU-004 | Database of the University of Münster - Biodiversity and Ecosystem Research Group's Vegetation Research in Western Siberia and Kazakhstan | 445 |  |
| AS-SA-001 | Vegetation Database of Saudi Arabia | 852 | ^28^ |
| AS-TJ-001 | Eastern Pamirs | 197 | ^29^ |
| AS-TR-002 | Vegetation Database of Oak Communities in Turkey | 1047 |  |
| AS-TW-001 | National Vegetation Database of Taiwan | 930 |  |
| AS-YE-001 | Socotra Vegetation Database | 331 | ^30^ |
| AU-AU-002 | AEKOS | 16421 | ^31^ |
| AU-NC-001 | New Caledonian Plant Inventory and Permanent Plot Network (NC-PIPPN) | 201 | ^32^ |
| AU-NZ-001 | New Zealand National Vegetation Databank | 1890 | ^33^ |
| AU-PG-001 | Forest Plots from Papua New Guinea | 63 | ^34^ |
| EU-00-002 | Nordic-Baltic Grassland Vegetation Database (NBGVD) | 2403 | ^35^ |
| EU-00-011 | Vegetation-Plot Database of the University of the Basque Country (BIOVEG) | 9661 | ^36^ |
| EU-00-013 | Balkan Dry Grasslands Database | 2429 | ^37^ |
| EU-00-016 | Mediterranean Ammophiletea Database | 4682 | ^38^ |
| EU-00-017 | European Coastal Vegetation Database | 1303 |  |
| EU-00-018 | The Nordic Vegetation Database | 1529 | ^39^ |
| EU-00-019 | Balkan Vegetation Database | 4007 | ^40^ |
| EU-00-020 | WetVegEurope | 12 | ^41^ |
| EU-00-022 | European Mire Vegetation Database | 2153 | ^42^ |
| EU-AL-001 | Vegetation Database of Albania | 110 | ^43^ |
| EU-AT-001 | Austrian Vegetation Database | 11580 | ^44^ |
| EU-BE-002 | INBOVEG | 5207 |  |
| EU-BG-001 | Bulgarian Vegetation Database | 1245 | ^45^ |
| EU-CH-005 | Swiss Forest Vegetation Database | 10584 | ^46^ |
| EU-CZ-001 | Czech National Phytosociological Database | 56659 | ^47^ |
| EU-DE-001 | VegMV | 17594 | ^48^ |
| EU-DE-013 | VegetWeb Germany | 4893 | ^49^ |
| EU-DE-014 | German Vegetation Reference Database (GVRD) | 22476 | ^50^ |
| EU-DK-002 | National Vegetation Database of Denmark | 16647 |  |
| EU-ES-001 | Iberian and Macaronesian Vegetation Information System (SIVIM) - Wetlands | 2281 |  |
| EU-FR-003 | SOPHY | 7193 | ^51^ |
| EU-GB-001 | UK National Vegetation Classification Database | 3220 |  |
| EU-GR-001 | KRITI | 155 |  |
| EU-GR-005 | Hellenic Natura 2000 Vegetation Database (HelNatVeg) | 4277 | ^52^ |
| EU-GR-006 | Hellenic Woodland Database | 636 | ^53^ |
| EU-HR-001 | Phytosociological Database of Non-Forest Vegetation in Croatia | 1966 | ^54^ |
| EU-HR-002 | Croatian Vegetation Database | 5777 |  |
| EU-HU-003 | CoenoDat Hungarian Phytosociological Database | 235 | ^55^ |
| EU-IT-001 | VegItaly | 5741 | ^56^ |
| EU-IT-010 | Vegetation database of Habitats in the Italian Alps - HabItAlp | 1970 | ^57^ |
| EU-IT-011 | Vegetation-Plot Database Sapienza University of Rome (VPD-Sapienza) | 8033 | ^58^ |
| EU-LT-001 | Lithuanian Vegetation Database | 1340 |  |
| EU-LV-001 | Semi-natural Grassland Vegetation Database of Latvia | 1497 | ^59^ |
| EU-MK-001 | Vegetation Database of the Republic of Macedonia | 210 |  |
| EU-NL-001 | Dutch National Vegetation Database | 35000 | ^60^ |
| EU-PL-001 | Polish Vegetation Database | 12679 | ^61^ |
| EU-RO-007 | Romanian Forest Database | 4641 | ^62^ |
| EU-RO-008 | Romanian Grassland Database | 1502 | ^63^ |
| EU-RS-002 | Vegetation Database Grassland Vegetation of Serbia | 3450 | ^64^ |
| EU-RU-002 | Lower Volga Valley Phytosociological Database | 844 | ^65^ |
| EU-RU-003 | Vegetation Database of the Volga and the Ural Rivers Basins | 705 | ^66^ |
| EU-RU-011 | Vegetation Database of Tatarstan | 1056 | ^67^ |
| EU-SI-001 | Vegetation Database of Slovenia | 5275 | ^68^ |
| EU-SK-001 | Slovak Vegetation Database | 20047 | ^69^ |
| EU-UA-001 | Ukrainian Grasslands Database | 3101 | ^70^ |
| EU-UA-006 | Vegetation Database of Ukraine and Adjacent Parts of Russia | 2259 |  |
| NA-00-002 | Tree Biodiversity Network (BIOTREE-NET) | 1756 | ^71^ |
| NA-CA-003 | Database of Timberline Vegetation in NW North America | 110 | ^72^ |
| NA-CA-004 | Understory of Sugar Maple Dominated Stands in Quebec and Ontario (Canada) | 150 | ^73^ |
| NA-CA-005 | Boreal Forest of Canada | 87 |  |
| NA-GL-001 | Vegetation Database of Greenland | 1 | ^74^ |
| NA-US-002 | VegBank | 13844 | ^75^ |
| NA-US-006 | Carolina Vegetation Survey Database | 13483 | ^76^ |
| NA-US-014 | Alaska-Arctic Vegetation Archive | 572 | ^77^ |
| SA-00-002 | VegPáramo | 1743 | ^78^ |
| SA-AR-002 | Vegetation Database of Central Argentina | 218 |  |
| SA-AR-003 | Argentina | 147 |  |
| SA-BO-003 | Bolivia Forest Plots | 75 |  |
| SA-BR-002 | Forest Inventory, State of Santa Catarina, Brazil (IFFSC Project) | 1669 | ^79^ |
| SA-CL-002 | SSAForests_Plots_db | 261 |  |
| SA-CL-003 | Chilean Park Transects - Fondecyt 1040528 | 160 | ^80^ |
| SA-CO-003 | Colombian Forest Plot Network (Col-Tree) | 207 | ^81^ |
| SA-EC-001 | Ecuador Forest Plot Database | 172 |  |
| SA-EC-002 | Galapagos veg | 85 |  |
| SA-UY-001 | Grassland relevés of Uruguay | 308 | ^82^ |
| - | Literature review | 1868 | Supplementary References 2 (^73-126^) |

## **Supplementary References 2 – Data Sources**

1 Lopez‐Gonzalez, G., Lewis, S. L., Burkitt, M. & Phillips, O. L. ForestPlots.net: a web application and research tool to manage and analyse tropical forest plot data. *J. Veg. Sci.* **22**, 610–613 (2011).

2 Chytrý, M. Database of Masaryk University Vegetation Research in Siberia. *Biodiver. Ecol.* **4**, 290 (2012).

3 Schmidt, M. *et al.* The West African Vegetation Database. *Biodiv. Ecol.* **4**, 105–110 (2012).

4 Muche, G., Schmiedel, U. & Jürgens, N. BIOTA Southern Africa Biodiversity Observatories Vegetation Database. *Biodiver. Ecol.* **4**, 111–123 (2012).

5 Revermann, R. *et al.* Vegetation database of the Okavango Basin. *Phytocoenologia* **46**, 103–104 (2016).

6 N'Guessan, A. E. *et al.* Drivers of biomass recovery in a secondary forested landscape of West Africa. *For. Ecol. Manag.* **433**, 325–331 (2019).

7 Müller, J. *Zur Vegetationsökologie der Savannenlandschaften im Sahel Burkina Fasos*, Frankfurt-Main Universität, (2003).

8 Kearsley, E. *et al.* Conventional tree height–diameter relationships significantly overestimate aboveground carbon stocks in the Central Congo Basin. *Nat. Comm.* **4**, 2269 (2013).

9 Djomo Nana, E. *et al.* Relationship between Survival Rate of Avian Artificial Nests and Forest Vegetation Structure along a Tropical Altitudinal Gradient on Mount Cameroon. *Biotropica* **47**, 758–764 (2015).

10 Wana, D. & Beierkuhnlein, C. Responses of plant functional types to environmental gradients in the south‐west Ethiopian highlands. *J. Trop. Ecol.* **27**, 289–304 (2011).

11 Finckh, M. Vegetation Database of Southern Morocco. *Biodiver. Ecol.* **4**, 297 (2012).

12 Strohbach, B. & Kangombe, F. National Phytosociological Database of Namibia. *Biodiver. Ecol.* **4**, 298–298 (2012).

13 Samimi, C. *Das Weidepotential im Gutu‐Distrikt (Zimbabwe) – Möglichkeiten und Grenzen der Modellierung unter Verwendung von Landsat TM‐5*. Vol. 19 (2003).

14 Černý, T. *et al.* Classification of Korean forests: patterns along geographic and environmental gradients. *Appl. Veg. Sci.* **18**, 5–22 (2015).

15 Nowak, A. *et al.* Vegetation of Middle Asia: the project state of the art after ten years of survey and future perspectives. *Phytocoenologia* **47**, 395–400 (2017).

16 Liu, H., Cui, H., Pott, R. & Speier, M. Vegetation of the woodland‐steppe ecotone in southeastern Inner Mongolia, China. *J. Veg. Sci.* **11**, 525–532 (2000).

17 Wang, Y. *et al.* Combined effects of livestock grazing and abiotic environment on vegetation and soils of grasslands across Tibet. *Appl. Veg. Sci.* **20**, 327–339 (2017).

18 Bruelheide, H. *et al.* Community assembly during secondary forest succession in a Chinese subtropical forest. *Ecol. Monogr.* **81**, 25–41 (2011).

19 Cheng, X.-L. *et al.* Taxonomic and phylogenetic diversity of vascular plants at Ma’anling volcano urban park in tropical Haikou, China: Reponses to soil properties. *PLoS One* **13**, e0198517 (2018).

20 Hatim, M. Vegetation Database of Sinai in Egypt. *Biodiver. Ecol.* **4**, 303 (2012).

21 Drescher, J. *et al.* Ecological and socio-economic functions across tropical land use systems after rainforest conversion. *Philos. Trans. R. Soc. Lond., B, Biol. Sci.* **371**, 20150275 (2016).

22 Dolezal, J., Dvorsky, M. & Kopecky, M. Vegetation dynamics at the upper elevational limit of vascular plants in Himalaya. *Sci. Rep.* **6**, 24881 (2016).

23 Borchardt, P. & Schickhoff, U. Vegetation Database of South‐Western Kyrgyzstan – the walnut‐wildfruit forests and alpine pastures. *Biodiver. Ecol.* **4**, 309 (2012).

24 Wagner, V. Eurosiberian meadows at their southern edge: patterns and phytogeography in the NW Tien Shan. *J. Veg. Sci.* **20**, 199–208 (2009).

25 von Wehrden, H., Wesche, K. & Miehe, G. Plant communities of the southern Mongolian Gobi. *Phytocoenologia* **39**, 331–376 (2009).

26 Chepinoga, V. V. Wetland Vegetation Database of Baikal Siberia (WETBS). *Biodiver. Ecol.* **4**, 311 (2012).

27 Korolyuk, A. *et al.* Database of Siberian Vegetation (DSV). *Biodiver. Ecol.* **4**, 312–312 (2012).

28 El-Sheikh, M. A. *et al.* SaudiVeg ecoinformatics: Aims, current status and perspectives. *Saudi J. Biol. Sci.* **24**, 389-398 (2017).

29 Vanselow, K. A. Eastern Pamirs – A vegetation‐plot database for the high mountain pastures of the Pamir Plateau (Tajikistan). *Phytocoenologia* **46**, 105 (2016).

30 De Sanctis, M. & Attorre, F. Socotra Vegetation Database. *Biodiver. Ecol.* **4**, 315 (2012).

31 Chabbi, A. & Loescher, H. W. *Terrestrial Ecosystem Research Infrastructures: Challenges and Opportunities*. (CRC Press, 2017).

32 Ibanez, T. *et al.* Structural and floristic diversity of mixed rainforest in New Caledonia: New data from the New Caledonian Plant Inventory and Permanent Plot Network (NC‐PIPPN. *Appl. Veg. Sci.* **17**, 386–397 (2014).

33 Wiser, S. K., Bellingham, P. J. & Burrows, L. E. Managing biodiversity information: development of New Zealand's National Vegetation Survey databank. *N. Z. J. Ecol.* **25**, 1–17 (2001).

34 Whitfeld, T. J. S. *et al.* Species richness, forest structure, and functional diversity during succession in the New Guinea lowlands. *Biotropica* **46**, 538–548 (2014).

35 Dengler, J. & Rūsiņa, S. Database dry grasslands in the Nordic and Baltic Region. *Biodiver. Ecol.* **4**, 319-320 (2012).

36 Biurrun, I., García-Mijangos, I., Campos, J. A., Herrera, M. & Loidi, J. Vegetation-plot database of the University of the Basque Country (BIOVEG). *Biodiver. Ecol.* **4**, 328 (2012).

37 Vassilev, K., Stevanović, Z. D., Cušterevska, R., Bergmeier, E. & Apostolova, I. Balkan Dry Grasslands Database. *Biodiver. Ecol.* **4**, 330–330 (2012).

38 Marcenò, C. & Jiménez‐Alfaro, B. The Mediterranean Ammophiletea Database: a comprehensive dataset of coastal dune vegetation. *Phytocoenologia* **47**, 95–105 (2017).

39 Lenoir, J. *et al.* Local temperatures inferred from plant communities suggest strong spatial buffering of climate warming across Northern Europe. *Glob. Change Biol.* **19**, 1470-1481 (2013).

40 Vassilev, K. *et al.* Balkan Vegetation Database: historical background, current status and future perspectives. *Phytocoenologia* **46**, 89–95 (2016).

41 Landucci, F. *et al.* WetVegEurope: a database of aquatic and wetland vegetation of Europe. *Phytocoenologia* **45**, 187-194 (2015).

42 Peterka, T., Jiroušek, M., Hájek, M. & Jiménez‐Alfaro, B. European Mire Vegetation Database: a gap‐oriented database for European fens and bogs. *Phytocoenologia* **45**, 291–297 (2015).

43 De Sanctis, M., Fanelli, G., Mullaj, A. & Attorre, F. Vegetation database of Albania. *Phytocoenologia* **47**, 107–108 (2017).

44 Willner, W., Berg, C. & Heiselmayer, P. Austrian Vegetation Database. *Biodiver. Ecol.* **4**, 333 (2012).

45 Apostolova, I., Sopotlieva, D., Pedashenko, H., Velev, N. & Vasilev, K. Bulgarian Vegetation Database: historic background, current status and future prospects. *Biodiver. Ecol.* **4**, 141–148 (2012).

46 Wohlgemuth, T. Swiss Forest Vegetation Database. *Biodiver. Ecol.* **4**, 340 (2012).

47 Chytrý, M. & Rafajová, M. Czech National Phytosociological Database: basic statistics of the available vegetation‐plot data. *Preslia* **75**, 1–15 (2003).

48 Jansen, F., Dengler, J. & Berg, C. VegMV – the vegetation database of Mecklenburg‐Vorpommern. *Biodiver. Ecol.* **4**, 149–160 (2012).

49 Ewald, J., May, R. & Kleikamp, M. VegetWeb – the national online‐repository of vegetation plots from Germany. *Biodiver. Ecol.* **4**, 173–175 (2012).

50 Jandt, U. & Bruelheide, H. German vegetation reference database (GVRD). *Biodiver. Ecol.* **4**, 355-355 (2012).

51 Garbolino, E., De Ruffray, P., Brisse, H. & Grandjouan, G. The phytosociological database SOPHY as the basis of plant socio-ecology and phytoclimatology in France. *Biodiver. Ecol.* **4**, 177–184 (2012).

52 Dimopoulos, P. & Tsiripidis, I. Hellenic Natura 2000 Vegetation Database (HelNAtVeg). *Biodiver. Ecol.* **4**, 388 (2012).

53 Fotiadis, G., Tsiripidis, I., Bergmeier, E. & Dimopoulos, P. Hellenic Woodland Database. *Biodiver. Ecol.* **4**, 389 (2012).

54 Stančić, Z. Phytosociological Database of Non‐Forest Vegetation in Croatia. *Biodiver. Ecol.* **4**, 391 (2012).

55 Lájer, K. *et al.* Hungarian Phytosociological database (COENODATREF): sampling methodology, nomenclature and its actual stage. *Annali di Botanica, Nuova Serie* **7**, 197–201 (2008).

56 Landucci, F. *et al.* VegItaly: The Italian collaborative project for a national vegetation database. *Plant Biosyst.* **146**, 756–763 (2012).

57 Casella, L., Bianco, P. M., Angelini, P. & Morroni, E. Italian National Vegetation Database (BVN/ISPRA). *Biodiver. Ecol.* **4**, 404 (2012).

58 Agrillo, E. *et al.* Nationwide Vegetation Plot Database – Sapienza University of Rome: state of the art, basic figures and future perspectives. *Phytocoenologia* **47**, 221–229 (2017).

59 Rūsiņa, S. Semi‐natural Grassland Vegetation Database of Latvia. *Biodiver. Ecol.* **4**, 409 (2012).

60 Schaminée, J. H. J. *et al.* *Schatten voor de natuur. Achtergronden, inventaris en toepassingen van de Landelijke Vegetatie Databank*. (KNNV Uitgeverij, 2006).

61 Kącki, Z. & Śliwiński, M. The Polish Vegetation Database: structure, resources and development. *Acta Soc. Bot. Pol.* **81**, 75–79 (2012).

62 Indreica, A., Turtureanu, P. D., Szabó, A. & Irimia, I. Romanian Forest Database: a phytosociological archive of woody vegetation. *Phytocoenologia* **47**, 389–393 (2017).

63 Vassilev, K. *et al.* The Romanian Grassland Database (RGD): historical background, current status and future perspectives. *Phytocoenologia* **48**, 91-100 (2018).

64 Aćić, S., Petrović, M., Dajić Stevanović, Z. & Šilc, U. Vegetation database Grassland vegetation in Serbia. *Biodiver. Ecol.* **4**, 418 (2012).

65 Golub, V. *et al.* Lower Volga Valley Phytosociological Database. *Biodiver. Ecol.* **4**, 419 (2012).

66 Lysenko, T., Kalmykova, O. & Mitroshenkova, A. Vegetation Database of the Volga and the Ural Rivers Basins. *Biodiver. Ecol.* **4**, 420–421 (2012).

67 Prokhorov, V., Rogova, T. & Kozhevnikova, M. Vegetation database of Tatarstan. *Phytocoenologia* **47**, 309–313 (2017).

68 Šilc, U. Vegetation Database of Slovenia. *Biodiver. Ecol.* **4**, 428 (2012).

69 Šibík, J. Slovak Vegetation Database. *Biodiver. Ecol.* **4**, 429 (2012).

70 Kuzemko, A. Ukrainian Grasslands Database. *Biodiver. Ecol.* **4**, 430 (2012).

71 Cayuela, L. *et al.* The Tree Biodiversity Network (BIOTREE-NET): prospects for biodiversity research and conservation in the Neotropics. *Biodiver. Ecol.* **4**, 211–224 (2012).

72 Wagner, V., Spribille, T., Abrahamczyk, S. & Bergmeier, E. Timberline meadows along a 1000 km transect in NW North America: species diversity and community patterns. *Appl. Veg. Sci.* **17**, 129–141 (2014).

73 Aubin, I., Gachet, S., Messier, C. & Bouchard, A. How resilient are northern hardwood forests to human disturbance? An evaluation using a plant functional group approach. *Ecoscience* **14**, 259-271 (2007).

74 Sieg, B., Drees, B. & Daniëls, F. J. A. Vegetation and altitudinal zonation in continental West Greenland. *Meddelelser om Grønland Bioscience* **57**, 1–93 (2006).

75 Peet, R. K., Lee, M. T., Jennings, M. D. & Faber-Langendoen, D. VegBank–a permanent, open-access archive for vegetation-plot data. *Biodiv. Ecol.* **4**, 233-241 (2012).

76 Peet, R. K. *et al.* Vegetation‐plot database of the Carolina Vegetation Survey. *Biodiver. Ecol.* **4**, 243–253 (2012).

77 Walker, D. A. *et al.* The Alaska Arctic Vegetation Archive (AVA‐AK. *Phytocoenologia* **46**, 221–229 (2016).

78 Peyre, G. *et al.* VegPáramo, a flora and vegetation database for the Andean páramo. *Phytocoenologia* **45**, 195-201 (2015).

79 Vibrans, A. C., Sevgnani, L., Lingner, D. V., Gasper, A. L. & Sabbagh, S. The Floristic and Forest Inventory of Santa Catarina State (IFFSC): methodological and operational aspects. *Pesquisa Florestal Brasileira* **30**, 291–302 (2010).

80 Pauchard, A., Fuentes, N., Jiménez, A., Bustamante, R. & Marticorena, A. *Alien plants homogenise protected areas: evidence from the landscape and regional scales in south central Chile* in *Plant Invasions in Protected Areas* (Springer, 2013).

81 González-Caro, S., Umaña, M. N., Álvarez, E., Stevenson, P. R. & Swenson, N. G. Phylogenetic alpha and beta diversity in tropical tree assemblages along regional-scale environmental gradients in northwest South America. *Journal of Plant Ecology* **7**, 145–153, (2014).

82 Bresciano, D., Altesor, A. & Rodríguez, C. The growth form of dominant grasses regulates the invasibility of Uruguayan grasslands. *Ecosphere* **5** (2014).
